# *Sequence*, a BioJS component for visualising sequences

**DOI:** 10.12688/f1000research.3-52.v1

**Published:** 2014-02-13

**Authors:** John Gomez, Rafael Jimenez

**Affiliations:** 1 European Bioinformatics Institute EMBL-EBI, Hinxton, CB10 1SD, UK

## Abstract

**Summary:** Sequences are probably the most common piece of information in sites providing biological data resources, particularly those related to genes and proteins. Multiple visual representations of the same sequence can be found across those sites. This can lead to an inconsistency compromising both the user experience and usability while working with graphical representations of a sequence. Furthermore, the code of the visualisation module is commonly embedded and merged with the rest of the application, making it difficult to reuse it in other applications. In this paper, we present a BioJS component for visualising sequences with a set of options supporting a flexible configuration of the visual representation, such as formats, colours, annotations, and columns, among others. This component aims to facilitate a common representation across different sites, making it easier for end users to move from one site to another.

**Availability:** http://www.ebi.ac.uk/Tools/biojs; http://dx.doi.org/10.5281/zenodo.8299

## Introduction

Visualising biological data on the web is a common practice on sites providing bio-oriented services and resources. A wide variety of JavaScript libraries are being used to build pieces of software capable of representing bio-entities such as DNA sequences ^1^, protein sequences ( http://www.uniprot.org), protein structures ( http://www.wwpdb.org), ontology trees ^2^, protein-protein interactions ( http://www.ebi.ac.uk/intact/) ^3^, and others. Therefore, a variety of possible visual representations for the same bio-entity can be found as a result of its multiple implementations. In many cases, such implementations are difficult to maintain, test, and reuse as they are developed only with one use case in mind. Furthermore, user experience (UX) and usability across different sites may be compromised.

**Figure 1.**
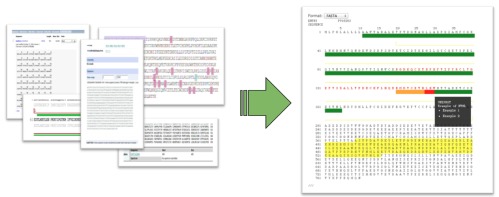
Multiple representations compiled as one flexible BioJS component.

One particular type of data commonly affected by multiple representations is the sequence, either a DNA or protein sequence. A sequence is a common bio-entity present in most sites offering biological data resources. Figure 1 shows different visual representations of a protein sequence as it can be found in Uniprot ( http://www.uniprot.org), Dasty ^4^ ( http://www.ebi.ac.uk/dasty) and Ensembl ( http://www.ensembl.org), among others ^5, 6^. Multiple features are identified across the entire set of sequences. Features such as formatting, indexing numbers, annotations, marks, colouring tags, and even the capability of user interaction are not integrated in one reusable piece of code. Instead, multiple representations prevail. Furthermore, web developers often make their own isolated efforts to reproduce those views for their sites and, in most cases, the representation is not identical, no documentation is available, and often they are not portable to other sites.

In this paper, a reusable component to visualise sequences is presented under the BioJS set of minimum standards for visualisation of biological components. BioJS is a community-driven standard to develop visualisation functionality ^7^. The library is developed using well-established methodologies and object-oriented design with inheritance that facilitates rapid development, reuse, extension, integration and deployment of web applications.

## The *Sequence* component

Exploring sequence visualisation across different sites reveals a set of features that should be supported by a single, reusable, and well documented piece of code, capable of painting sequences on the web in a consistent manner. In this sense, BioJS provides a baseline for Javascript coding and development to create pieces of reusable code, called components. Creating a new *Sequence* component consists of extending a core BioJS class and defining three core concepts: options, methods and events. Options are the data required by the component for initialisation, while methods and events are actions supported in execution time. Methods are fired externally while events are triggered in the component and exposed to external listeners.

**Figure 2.**
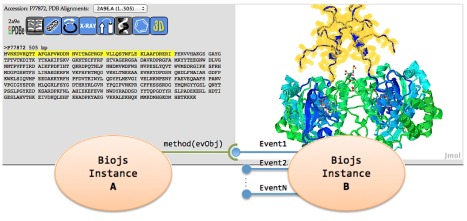
Example of communication between Sequence and Protein3D components.

**Figure 3.**
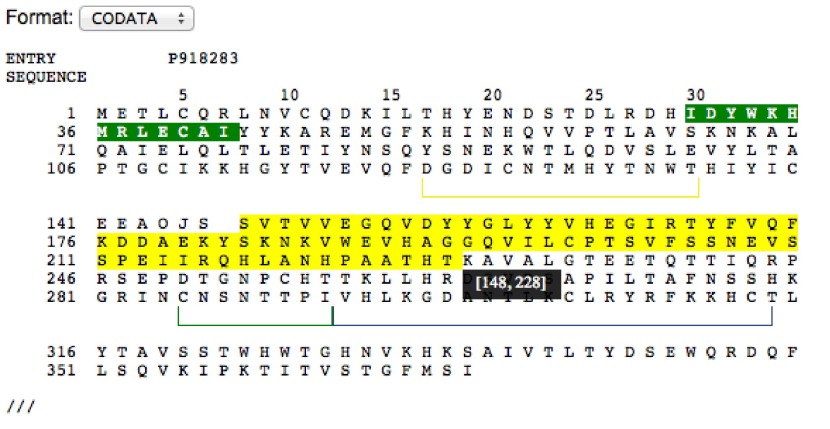
Example displaying the sequence corresponding to the UniProt accession P918283. The part highlighted in yellow denotes the current selection, the black pop-up box indicates what the interval is with every move of the pointer. Green highlight denotes an annotation on that interval. Multiple annotations are supported.

Methods and events allow the component to communicate with others components as well as web applications. Figure 2 shows a working example implemented within the Biotea project ^8^. This example shows a communication between two component instances, the *Sequence* component and the *Protein3D* component. When a region (highlighted in yellow) on the sequence is selected, automatically a selection action is fired in the Protein3D. Additionally, *Sequence* supports a set of options to change the visual representation of the sequence by using different formats, colours, indexing numbers, annotations and more. It helps deployment because the component can be easily fitted to the particular need. Figure 3 shows an example of the *Sequence* component displaying the protein P918283 in CODATA format.

As any other BioJS component, the *Sequence* component is well documented and has been tested during development, not only for functionality but also for usability. BioJS makes it easier to document the code by adding annotations that are later exposed as a web page. Thus, human-friendly documentation is generated without any additional effort. BioJS web pages for components are compiled in a registry that acts as a showcase of working examples extracted from the component annotations. The registry makes it easier for both developers and end users to understand components and their functionality. Once a component has met the BioJS guidelines, it becomes a candidate to be submitted and publicly shared in the common repository of components, the EBI BioJS registry ( http://www.ebi.ac.uk/Tools/biojs/registry/). There, it is possible to find more information about options, installation, methods, and events ( http://www.ebi.ac.uk/Tools/biojs/registry/Biojs.Sequence.html).

## Future work

Currently, the *Sequence* component supports the visualisation of a single strand. However, in some cases, it should be more interesting to display similarities between two or multiple sequences. Another possible extension is using this component as a base for multiple aligned sequences visualisation. Aligner algorithms ^9^ could be run on the server side or consumed from a web service ^10^ while the component would be in charge of painting the similarities, taking advantage of already developed features such as colouring, highlighting, and tagging.

Collaborative work and social networking is nowadays a mechanism for knowledge construction. Such features can be integrated into the *Sequence* component so end users can submit sequences and annotations to public sequence databases such as UniProt. Comments and references could also be added, adding valuable information for a researcher during his/her investigation.

## Software availability

Zenodo: Sequence BioJS component for visualising sequences, doi: 10.5281/zenodo.8299 ^11^.

GitHuB: BioJS, http://www.ebi.ac.uk/Tools/biojs.
