# Supplementary material for: Sequence, a BioJS component for visualising sequences
Source: F1000Res. 2014 Feb 13;3:52. [Version 1] doi: 10.12688/f1000research.3-52.v1 (PMC4103491; doi:10.12688/f1000research.3-52.v1)
Supplement: Peer review history

# Peer review: Referee response for version 1

**DOI:** 10.5256/f1000research.3717.r3802

**Published:** 2014-03-17

**Authors:** Jeremy Goecks

**Affiliations:** 1 Computational Biology Institute, George Washington University, Washington, DC, USA

Here, the authors present *Sequence*, a web-based visualization component for biological sequence data implemented in JavaScript. Investigators can use *Sequence* to visualize both DNA and protein sequences, either as a standalone visualization or together with other visualizations.

Strengths of *Sequence* include (a) the ability to customize sequence using options and (b) integration of sequence via events. These features ensure that *Sequence* can be used in a wide variety of applications.

What is missing from this manuscript is a description of how well *Sequence* scales to large sequences and whether a *Sequence* visualization can be updated dynamically in response to events from other components.

Overall *, Sequence* is a solid contribution to web-based visualization that is useful as it is and forms the foundation for more complex web-based sequence visualization in the future.

I have read this submission. I believe that I have an appropriate level of expertise to confirm that it is of an acceptable scientific standard.

# Peer review: Referee response for version 1

**DOI:** 10.5256/f1000research.3717.r3694

**Published:** 2014-03-17

**Authors:** Christoph Gille

**Affiliations:** 1 Computational Biochemistry Group, Institute of Biochemistry, University Medicine Berlin (Charité), Berlin, Germany

**General**

The authors present the first re-usable JavaScript based sequence component. It can be used in web applications dealing with bio-polymers like proteins and nucleotide sequences and can also interact with other parts of the website via events. 

Previously, Java applets have been used for interactive web content. However, Java constitutes an additional layer of software and thereby carries an own set of technical problems and risks. For this reason,

JavaScript is being increasingly used at the client side. In this respect, the development of BioJS components follows a general trend.

The BioJS registry is the first and only framework plus standard  for interactive web components, and *Sequence* will be one of the most important components following the BioJS specification. Therefore, I expect that the *Sequence* component will be widely used in bioinformatics web services. Even if current features might not satisfy all needs, the BioJS format allows for extensions and incorporation of new features with the source code clear and well documented, allowing developers to change it to their requirements.

**Manuscript**

I would suggest replacing the word "compiled" with another word (in the figure 1 legend and in the third paragraph of the *Sequence *component section) as it might be mistaken for source code getting compiled on a server like on the Debian Linux server.

The manuscript does not provide answer to some important questions:

1. Is the length of the sequence limited?
2. Is the sequence immutable? Or could it change like alternative splicing? Can parts of the sequences be hidden like cutting of signal peptide?
3. "Indexing numbers" - does the numbering support PDB insertion codes?

It would be good if these points could be clarified in the manuscript.

**Example**

For demonstration, the authors have coupled the sequence view with a BioJS 3D component.

With the newest Java, the JMol applet fails to start with the message: " *Your security system has blocked an untrusted ...*". I expect that the line Permissions: sandbox in the jar-file manifest and signing the jar-file will fix the problem.

The authors should also consider using a JavaScript based 3D visualization.

**API**

On events like 'Annotation Clicked', there is no parameter indicating whether the context pop-up trigger (right click, long touch) is active and what modifier keys like Shift and Ctrl are pressed - this should be made clearer for ease of use.

I have read this submission. I believe that I have an appropriate level of expertise to confirm that it is of an acceptable scientific standard.
